# Constitutive Expression of *Thermobifida fusca* Thermostable Acetylxylan Esterase Gene in *Pichia pastoris*

**DOI:** 10.3390/ijms11125143

**Published:** 2010-12-15

**Authors:** Chao-Hsun Yang, Kun-I Lin, Gen-Hung Chen, Yu-Fen Chen, Cheng-Yu Chen, Wei-Lin Chen, Yu-Chun Huang

**Affiliations:** 1 Department of Cosmetic Science, Providence University, 200, Chung-Chi Rd., Taichung, 43301, Taiwan; E-Mails chyang@pu.edu.tw (C.-H.Y.); ghchen2@pu.edu.tw (G.-H.C); yfchen@pu.edu.tw (Y.-F.C.); zychan1268@gmail.com (C.-Y.C); wei.lin46@gmail.com (W.-L.C); 2 Department of Obstetrics & Gynecology, Chang Bing Show Chwan Memorial Hospital, No. 6 Lugong Rd. Lukang Zhen, Changhua County, Taiwan; E-Mail: konyi@xuite.net

**Keywords:** acetylxylan esterase (AXE), Pichia pastoris, Thermobifida fusca, constitutive expression

## Abstract

A gene encoding the thermostable acetylxylan esterase (AXE) in *Thermobifida fusca* NTU22 was amplified by PCR, sequenced and cloned into the *Pichia pastoris* X-33 host strain using the vector pGAPZαA, allowing constitutive expression and secretion of the protein. Recombinant expression resulted in high levels of extracellular AXE production, as high as 526 U/mL in the Hinton flask culture broth. The purified enzyme showed a single band at about 28 kDa by SDS-polyacrylamide gel electrophoresis after being treated with endo-β-*N*-acetylglycosaminidase H; this agrees with the predicted size based on the nucleotide sequence. About 70% of the original activity remained after heat treatment at 60 °C for three hours. The optimal pH and temperature of the purified enzyme were 8.0 and 60 °C, respectively. The properties of the purified AXE from the *P. pastoris* transformant are similar to those of the AXE from an *E. coli* transformant.

## 1. Introduction

Xylan is a major hemicellulose component of plants and is the most abundant renewable polysaccharide in nature. The complex composition and structure of xylan varies across different plant species [1,2]. It is composed of d-xylopyranosyl residues joined by β-1,4-glycosidic linkages. Some of these xylosyl residues can be substituted at the C2 and/or C3 positions with acetic acid, 4-*O*-methylglucuronic acid and arabinose [3]. It is not surprising that the synergistic actions of a consortium of microbial enzymes are required for the complete degradation of xylan. Endo-β-1,4-xylanase (EC 3.2.1.8) can randomly cleave the polysaccharide backbone into shorter xylooligosaccharides. These xylooligosaccharides are further degraded by other glycoside hydrolases and esterases [1,2]. Acetylxylan and xylooligosaccharides are deesterified by acetylxylan esterases (EC 3.1.1.72). These enzymes have been found and isolated from many genera, including *Trichoderma* [4], *Aspergillus* [5], *Penicillium* [6], *Schizophyllum* [7], *Rhodotorula* [8], *Streptomyces* [9], *Fibrobacter* [10], *Bacillus* [11], *Hypocrea* [12], *Volvariella* [13], *Neocallimastix* [14] and *Thermobifida* [15].

The importance and development of industrial biotechnology processing has led to the utilization of microbial enzymes in various applications. To produce enzymes for the development of enzymatic degradation of renewable lignocelluloses, we have isolated a potent extracellular lignocellulolytic enzyme-producing thermophilic actinomycete, *Thermobifida fusca* NTU22, from compost soils collected in Taiwan [16]. Interestingly, it has been reported that *T. fusca* can produce intracellular and extracellular acetylxylan esterases (AXEs) on oat-spelt xylan [15]. One of the extracellular AXEs was purified and some of its properties were discussed. The molecular mass of the purified enzyme was estimated to be 30 kDa by SDS-PAGE and 28 kDa by gel filtration on Sepharose CL-6B, indicating that the AXE from *T. fusca* NTU22 is a monomer. The pI value of the purified enzyme was estimated to be 6.55 by isoelectric focusing gel electrophoresis. The N-terminal amino acid sequence of the purified esterase was ANPYERGP. The optimum pH and temperature for the purified enzyme were 8.0 and 80 °C, respectively. Zn^2+^, Hg^2+^, PMSF and DIPF inhibited the enzyme activity. The Km value for *p*-nitrophenyl acetate and acetylxylan were 1.86 μM and 0.15%, respectively [17]. The *axe* gene, which encodes the AXE from *T. fusca* NTU22, was cloned, sequenced and expressed in *Escherichia coli* (Accession No. HM 193859). The gene consists of 786 base pairs and encodes a protein of 262 amino acids. The G + C content of the *axe* coding sequence was 66.9% and the protein had a predicted isoelectric point of 6.34. The deduced amino acid sequence of the AXE exhibited a high degree of similarity with BTA-hydrolase from *T. fusca* DSM43793, esterase from *Thermobifida alba* and lipase from *Streptomyces albus* [18].

In the light of economic benefits, several thermostable enzyme genes from thermophilic microorganisms have been cloned and expressed in mesophilic microorganisms to reduce the energy needed for cultivation [19,20]. *E. coli* is the first choice for heterologous protein expression due to the ease of genetic manipulation, availability of efficient genetic tools, high transformation efficiency and rapid growth rates. However, misfolding and intracellular production often presents the risk of inclusion body formation. In this study, we expressed the AXE gene from *T. fusca* NTU22 in a host system other than *E. coli* because of its potential application as a food supplement. Recently, among many mesophilic host systems, *Pichia pastoris*, methylotrophic yeast capable of performing many eukaryotic posttranslational modifications, has been considered as an excellent host system for heterologous proteins expression. It is well known that expression can be driven by the strong alcohol oxidase I (*AOX1*) promoter under methanol induction [21]. In addition, the glyceraldehyde-3-phosphate dehydrogenase (*GAP*) promoter has been used for constitutive expression of several heterologous proteins without methanol induction in *P. pastoris* [14].

The aim of this study was to constitutively express the AXE gene (*axe*) from the thermophilic actinomycetes, *T. fusca* NTU22, in *P. pastoris* X-33. Some properties of the heterologous expressed enzyme were also investigated.

## 2. Results and Discussion

### 2.1. Amplification and Construction of the AXE Gene in *P. pastoris*

In order to amplify the AXE gene from plasmid ER405-6, two primers were designed for PCR. According to agarose gel electrophoresis of the PCR product, one major band was apparent at approximately 800 bp and could be eluted for further processes. The *axe* coding sequence was cloned into the pGAPZαA vector using the *Xho*I/*Xba*I restriction sites as described earlier. This construction allowed *axe* coding sequence to be theoretically in-frame with the α-factor secretion signal in pGAPZαA. Following a sequence check, the construct, denoted as pGAPZ-*axe*, was *Bgl*II-linearized and electroporated into *P. pastoris* X-33. Four transformants were selected for high resistance to Zeocin (2,000 μg/mL). The genomic PCR assay revealed that all the transformants contained an integrated *axe* coding sequence in the genomic DNA. Among these four transformants, the transformant (pGAPZ-*axe*) could produce the highest AXE activity and was selected for further experiments.

### 2.2. Expression of the AXE Gene (*axe*) in *P. pastoris*

**Figure 1.**
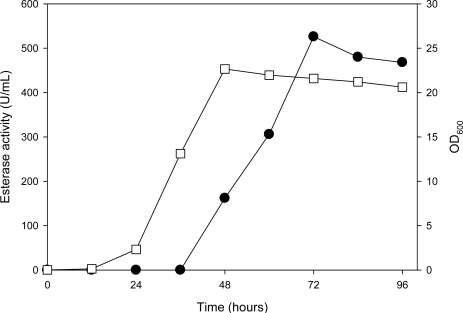
Time course for the expression of AXE activity by *P. pastoris* transformant (pGAPZ-*axe*). Cells were grown aerobically in a 500 mL Hinton flask loaded with 50 mL of medium consisting of YPD broth, and were incubated at 28 °C, 150 rpm for 96 hours. (•), esterase activity; (□), OD_600_.

The fermentation conditions for constitutive expression of the AXE were investigated in a 500 mL Hinton flask loaded with 50 mL YPD broth at 28 °C. Transformant (pGAPZ-*axe*) grew logarithmically from 24 to 48 hours and then entered a stationary phase (Figure 1). The biomass reached approximately 22 of the OD_600_ value after 48-hours incubation. The enzyme production pattern in this transformant indicated that AXE synthesis began in the middle logarithmic phase and continued to be produced during the stationary phase where the maximum activity (526 U/mL) was reached in the culture broth. The AXE activity was about 500-times higher than that observed when expressed from an *E. coli* transformant [18]. The control strain, *P. pastoris* (pGAPZαA), could also produce esterase activity under the same culture conditions (data not shown). In the *GAP* promoter expression system, the cloned heterologous protein will be expressed along with cell growth if the protein is not toxic for the cell [23,24]. Such a result was observed in this study. As shown in Figure 1, the rapid production of the extracellular AXE occurred in parallel with an increase in biomass accumulation of the *P. pastoris* transformant.

### 2.3. Purification of AXE from *P. pastoris* Transformant

The purification of AXE was performed as described in the Experimental Section. The results of the total purification are summarized in Table 1. The purified enzyme exhibited 0.68% of the total initial activity and there was a 26.1-fold increase in specific activity when compared with the crude culture filtrate. The amount of extracellular proteins in the culture broth of the *P. pastoris* transformant was fewer than that in the cell free extract of the *E. coli* transformant. This will facilitate the application of the enzyme in an industrial process immediately without complicated purification procedures. Most of the non-specific esterase activity from the *P. pastoris* host was lost in the ultrafiltration process, resulting in a decrease of the purification fold.

### 2.4. Properties of AXE from *P. pastoris* Transformant

**Figure 2.**
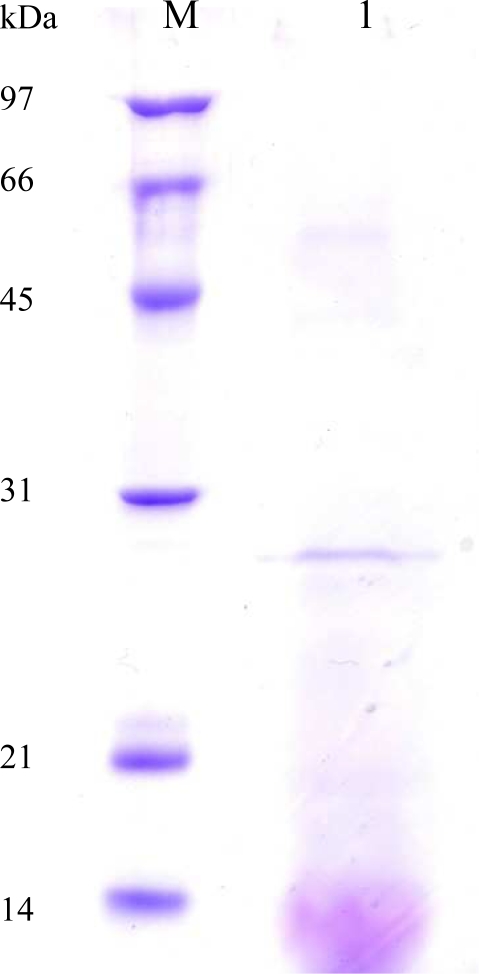
SDS-PAGE of purified protein sample containing AXE from *P. pastoris* transformant (*axe*). Lane M = molecular weight markers; 1 = protein sample containing AXE from *P. pastoris* transformant.

As shown in Figure 2, after being treated with endo-β-*N*-acetylglycosaminidase H for deglycosylation, the purified enzyme showed an apparent single protein band on SDS-PAGE (10% gel). The subunit size of the single protein band was estimated to be 28 kDa from its mobility relative to standard proteins by SDS-PAGE. The optimal pH and temperature of the AXE from *P. pastoris* transformant (pGAPZ-*axe*) were 8.0 and 60 °C, respectively. About 70% of the original AXE activity remained after heat treatment at 60 °C for three hours. Yang *et al*. [17] reported that 70% of the AXE activity from *T. fusca* NTU22 remained after heat treatment at 70 °C for three hours. Apparently the thermostability of the AXE from the *P. pastoris* transformant is lower than the enzyme from the original strain, *T. fusca* NTU22.

## 3. Experimental Section

### 3.1. Microorganisms and Vectors

Thermophilic actinomycetes, *T. fusca* NTU22, which was isolated from compost soils collected in Taiwan, was used in this study [16]. *P. pastoris* X-33 and pGAPZαA were purchased from Invitrogen (Sandiego, CA, USA). The plasmid propagation in the expression work was accomplished with *E. coli* Top 10 (Invitrogen, Sandiego, CA, USA). The plasmid ER405-6 was constructed with the pGEM^®^-T Easy Vector and a 800 bp inserted fragment, which possessed an AXE gene *axe* [18].

### 3.2. Materials

Yeast extract, peptone, tryptone and agar were purchased from BD (Sparks, MD, USA). Zeocin was obtained from Cayla (Toulouse, France). Restriction endonucleases and T4 DNA ligation kit were purchased from Roche (Mannheim, Germany). For polymerase chain reactions, the Vio Twin Pack Kits comprising VioTag DNA polymerase, polymerase chain reaction buffer and deoxynucleotides were obtained from Viogene (Sunnyvale, CA, USA). Ni-Sepharose™ High Performance resin was purchased from GE Healthcare (Little Chalfont, UK). PiNK Prestained Protein Ladder was purchased from GeneDirex (Las Vegas, NV, USA). The protein assay kit was obtained from Bio-Rad Laboratories (Hercules, CA, USA). Endo-β-*N*-acetylcosaminidase H was purchased from New England Biolabs Inc. (Beverly, MA, USA). Inorganic salts and all other chemicals were purchased from Sigma (St. Louis, MO, USA).

### 3.3. Construction of the Amylase Expression Plasmid

The *T. fusca* NTU22 AXE gene was amplified using the primers 5′-AATTCTAGACCAAATGGACAGGTGCTGCGATACTCTTC-3′ (*Xho*I site is underlined) and 5′-AACTCGAGAAACGTGAGGCTGAAGCAGCGGCGAATCCGTATGAACGG-3′ (*Xba*I site is underlined), using ER405-6 as the template. The amplification was performed using a DNA thermal cycler (Perkin Elmer, MA, USA) with the following conditions: the first step was initiated at 95 °C for 5 min, followed by 30 cycles of 95 °C for 30 s, 65 °C for 30 s and 72 °C for 90 s, and the final extension was carried out at 72 °C for 7 min. The gel-purified PCR product was digested with *Xho*I and *Xba*I and cloned into *Xho*I and *Xba*I digested pGAPZαA. After being transformed into *E. coli* Top 10, one recombinant plasmid designated as pGAPZα-*axe* was selected on low salt LB agar plates (5 g/L yeast extract, 10 g/L tryptone, 5 g/L NaCl, 15 g/L agar, and adjusted pH to 7.5) containing 25 μg/mL Zeocin. The proper insert orientation was checked by restriction analysis and sequencing as described above [25].

### 3.4. Transformation and Screening of *P. pastoris*

Ten micrograms of recombinant plasmid (pGAPZα-*axe*) was linearized with *Bgl*II, and electroporated into *P. pastoris* X-33 using the following conditions: 1.5 kV, 25 μF, 200 ohm and 5 ms, using a GenePulser (Bio-Rad, CA, USA). The transformants were selected at 28 °C on YPDS agar plates (10 g/L yeast extract, 20 g/L peptone, 20 g/L dextrose, 20 g/L agar and 1 M sorbitol) containing 100 μg/mL Zeocin for 4–6 days. Screening for high level expression transformants was done by replicating the colonies obtained from the YPDS agar plates containing 100 μg/mL Zeocin onto YPDS agar plates with a higher Zeocin concentration (2,000 μg/mL). Transformants with higher Zeocin-resistance were obtained and checked for the integration of the construct into the *P. pastoris* X-33 genome by genomic PCR [26].

### 3.5. Biomass and Esterase Activity Assay

Biomass production of the *P. pastoris* transformant was evaluated by measuring the optical density at 600 nm (OD_600_ value). Esterase activity was assayed as described by Kodemi *et al*. [27] with slight modification. We used spectrophotometric analysis with *p*-nitrophenyl acetate as a substrate; the release of *p*-nitrophenol was determined at 405 nm. One unit of enzyme activity was defined as the amount of the enzyme releasing 1 μmol of *p*-nitrophenol per min at 25 °C, pH 8.0.

### 3.6. Cultivation and Expression of AXE in Hinton Flask

The transformant resistant to high concentration of Zeocin was incubated in 50 mL YPD broth (10 g/L yeast extract, 20 g/L peptone, 20 g/L dextrose) in 500 mL Hinton flasks and shaken (150 rpm) at 28 °C. After the cultivation was carried out for several days, the culture broth was centrifuged at 10,000 × *g* for 30 min at 4 °C and finally the supernatant was tested for AXE activity.

### 3.7. Enzyme Purification

All purification procedures were done at 4 °C in 20 mM Tris-HCl buffer (pH 7.5) unless otherwise stated. After 72-hours of cultivation of the *P. pastoris* transformant (pGAPZα-*axe*) in a 500 mL Hinton flask, the fermentation broth was centrifuged at 3,000 × g for 30 min to remove cells. The supernatant was then applied to a Ni-Sepharose™ High Performance column (1.13 cm × 8 cm) pre-equilibrated with Tris-HCl buffer. After washing with wash buffer (20 mM Tris-HCl, 500 mM sodium chloride, 40 mM imidazole; pH 7.5) to remove the unbound protein, the enzyme was eluted with elution buffer (20 mM Tris-HCl, 500 mM sodium chloride, 500 mM imidazole; pH 7.5). The eluted enzymatically active fractions were pooled and used as the purified enzyme.

### 3.8. SDS-Polyacrylamide Gel Electrophoresis (SDS-PAGE)

The molecular mass of the purified enzyme was determined by using SDS-PAGE (10% polyacrylamide). PiNK Prestained Protein Ladder was used as molecular mass standards. The electrophoresis was carried out at 150 V for 1 h. The gel was stained with 0.27% Coomassie Brilliant Blue R-250, and destained by washing overnight with a mixture of acetic acid-methanol-water (10:20:70, V/V).

### 3.9. Deglycosylation of AXE from *P. pastoris* Transformant

The purified enzyme from *P. pastoris* transformant was deglycosylated by denaturing the glycoprotein at 100 °C for 10 min, and then endo-β-*N*-acetylglycos-aminidase H was added to perform deglycosylation at 37 °C for 1 h. All manipulations followed the manufacturer’s instructions.

## 4. Conclusions

We are interested in the production of lignocellulolytic enzyme from local thermophilic actinomycetes to explore their use in novel biotechnological applications. The thermostable AXE gene (*axe*) from thermophilic actinomycetes, *T. fusca* NTU22, was heterologously expressed in the mesophilic host system, *P. pastoris*, for further exploration. This *GAP* expression system is more suitable than the *AOX* expression system for large-scale production because the hazard and cost associated with the storage and delivery of large volumes of methanol are eliminated. Thus, the features of the *GAP* expression system may contribute significantly to the development of cost-effective methods for large-scale production of heterologous recombinant proteins. These findings hold promise in large-scale production of thermostable AXE and application in both agricultural and food industries.

## Figures and Tables

**Table 1.** Summary of the purification of AXE from *P. pastoris* transformant (*axe*).

**0**	**Total activity (U)**	**Total protein (mg)**	**Specific activity (U/mg)**	**Purification (fold)**	**Yield (%)**
Culture	469,698.8	637.1	737.2	1.0	100.0
Ultrafiltration	32,729.1	283.5	115.4	0.2	6.97
Ni-Sepharose™	3,214.0	0.167	19,245.5	26.1	0.68
